# Evaluation of the Effects of *Dorema aucheri* (Bilhar) Hydroalcoholic Extract on Wound Healing of Streptozotocin-Induced Diabetic Rats: A Stereological Study

**DOI:** 10.1155/ije/8278940

**Published:** 2025-11-16

**Authors:** Forough Saki, Aliakbar Banihashemi, Farhad Koohpeyma, Sanaz Dastghaib, Alireza Raeisi, Mesbah Shams

**Affiliations:** ^1^Endocrinology and Metabolism Research Center, Shiraz University of Medical Science, Shiraz, Iran; ^2^Student Research Committee, Shiraz University of Medical Sciences, Shiraz, Iran; ^3^Autophagy Research Center, Shiraz University of Medical Sciences, Shiraz, Iran

**Keywords:** diabetes, *Dorema aucheri*, *rat*, stereology, wound healing

## Abstract

**Background and Purpose:**

One of the most common complications of diabetes is impaired healing of diabetic wounds. Given the antidiabetic and antimicrobial effects of the *Dorema aucheri* plant, the present study was conducted to determine the impact of the alcoholic extract of this plant on the healing of full-thickness diabetic wounds in male rats.

**Materials and Methods:**

In this experimental study, 48 male rats were randomly divided into 6 groups: control wound, diabetic wound, diabetic wound with carboxymethylcellulose base gel, and diabetic wounds with the combination of *D. aucheri* extract with percentages of 2%, 5%, and 10%. The wound in all groups was the full thickness of the skin. The drugs were administered topically, once daily, for 14 days. Also, to measure the percentage of wound healing, we took photographs of the samples on Days 0, 3, 7, and 14. Finally, skin samples were taken from the wound repair site for stereological evaluation.

**Results:**

In comparison to the other groups, the group treated with 5% *D. aucheri* extract showed a better healing speed, volume of re-epithelialization, density of collagen deposition, and neovascularization (*p* ≤ 0.05). The fibroblast number density, collagen density, and hair follicle volume densities were all lower in the 10% *D. aucheri*-treated group than in the other groups (*p* ≤ 0.001).

**Conclusion:**

The hydroalcoholic extract of the *D. aucheri* plant, particularly at a concentration of 5%, has beneficial effects on the healing process of diabetic wounds.

## 1. Introduction

Wound healing is the result of a set of processes that ultimately lead to simulating the lost tissue as much as possible [1, 2]. It is a natural reparative response to tissue damage, which can be divided into four overlapping stages that begin with homeostasis; inflammation, proliferation, and maturation are the other stages, which finally end with scar formation [3]. This process is a sequence of regulated biological processes, including inflammation, fibroplasia, angiogenesis, formation of new collagen fibers, epithelialization, migration of different types of cells, formation of granule tissue, and wound contraction [4]. These processes require a coordinated interaction between inflammatory cells, fibroblasts, keratinocytes, biochemical mediators, and extracellular matrix (ECM) molecules [5]. Meanwhile, some underlying diseases disrupt the natural healing process and cause a delay in wound closure and chronicity, one of the most important of which is diabetes mellitus [6].

Diabetes includes a group of common metabolic disorders, the common denominator of all of which is hyperglycemia [7]. The global prevalence of diabetes has increased significantly during the last decade. In the United States, an annual cost of over one billion dollars and over 70,000 lower limb amputations in diabetic patients are imposed on the health system [8]. In Iran, according to the latest statistics, about 510,000 individuals are exposed to lower limb wounds and amputations due to diabetes [9]. Complications of diabetes affect many organs of the body and are responsible for most of the morbidity and mortality caused by diabetes [10, 11].

Diabetic ulcers are one of the most important complications of diabetes. It is the most common cause of nontraumatic amputation of lower limbs in the world. These foot ulcers and infections are one of the main causes of disability in patients with diabetes [12]. Today, despite the implementation of preventive methods, foot ulcers and infections are common and cause serious problems for diabetic patients [13]. Atherosclerosis and peripheral neuropathy are the underlying mechanisms related to the occurrence of ulcers [14]. Diabetic people are more prone to capillary basement membrane thickening, arterial hyalinosis, and endothelial proliferation [15]. Skin wounds and the reduction in their healing time are one of the most important aspects of medical sciences. The process of wound healing depends on such factors as the production, storage, and binding of collagen and the functioning of skin tissue cells.

In chronic ulcers, the presence of necrotic tissue, inadequate perfusion, and inflammation all slow down the healing process. Because it is warm, moist, and nutrient-rich, it may be the perfect environment for microbes to grow [16]. In this context, it is imperative to prepare a wound dressing with antibacterial properties. Additionally, the usage of highly hydrated hydrogels that have the capacity to trap water makes these materials viable substitutes for wound healing applications [17]. Since it can be cross-linked to create biodegradable hydrogel, carboxymethyl cellulose (CMC), a water-soluble semisynthetic ether derivative of cellulose, has been utilized in drug carriers for wound care because of its high water absorption capacity and favorable biocompatibility [18, 19]. CMC is utilized as an ingredient in interactive dressings, particularly hydrocolloid dressings, for wound healing. These dressings collect wound exudate and create a moist environment, which aids in tissue repair. Burn and surgical wounds can benefit from CMC's ability to lessen pain and inflammation. CMC is very appealing for the creation of wound dressings due to its qualities as a nontoxic and biocompatible polymer with bone and skin [20, 21]. In countries like India, China, and Iran, where traditional medicine has a long history, there is important information about the use of many unknown plants in the treatment of wounds [22]. Approaching natural substances to produce new and more effective drugs is more compatible with the body of living organisms and has fewer side effects; it has been included in the agenda of pharmaceutical science and human medicine researchers [23].

Belonging to the Apiaceae family, *Dorema aucheri* (Bilhar) is a medicinal plant utilized extensively in Iranian traditional medicine due to its expectorant and anti-inflammatory qualities. It grows in the chilly highlands of Fars, Kohgiluyeh & Boyer-Ahmad, and Isfahan provinces in the early spring [24]. Despite being a medicinal species in the Apiaceae family, *D. aucheri* is primarily used as a condiment in cooking. It is known by several interchangeable scientific names, such as *Ferula aucheri*, *Angelica dura* K. Koch, *Dorema robustum*, and *D. aucheri Boiss*. The taxonomic categorization of this plant is as follows: Kingdom: Plantae, Clade: Tracheophytes, Clade: Angiosperms, Clade: Eudicots, Clade: Asterids, Order: Apiales, Family: Apiaceae, Genus: *Dorema*, Species: *D. aucheri*. The chief biologically active metabolites of this plant extract include essential oil, resin, coumarins, furanocoumarins, all kinds of terpenes, three saponins, acetylene components, flavonoids, and a small amount of alkaloids [25]. Following GC and GC/MS analysis of the extracted products, the primary constituents of *D. aucheri* essential oils were found to include cuprene, β-gurgunene, α-gurjunene, carvacrol, thymol, α-pinene, p-cymene, limonene, and β-caryophyllene [26, 27]. Furthermore, δ-cadinene and α-odesmol were the most prevalent main bioactive flavonoid compounds isolated from *D. aucheri* [28, 29]. It has been experimentally shown that this plant has analgesic, anti-inflammatory, and antibacterial properties. Also, due to the presence of many flavonoids in this plant and the presence of polyphenols and several other biologically active products, it has been determined that this plant plays a role in reducing free radicals in the plasma. Researchers have demonstrated the plant's antibacterial, antilipid peroxidation, antidiabetic, and antioxidant qualities (including total phenolic, total flavonoids, total anthocyanins, and others) [30–34]. Additionally, based on its phytochemical content (polysaccharides, phenolics, and flavonoids), *D. aucheri* gum has been shown to promote wound healing. The accelerated wound healing process in mice following topical application of *D. aucheri* gum is mediated by positive modulation of angiogenesis and neovascularization, tissue antioxidants, and inflammatory cytokines [35]. Given the well-documented anti-inflammatory, antimicrobial, and antioxidant properties of *D. aucheri*, the primary aim of this study was to evaluate the effect of *D. aucheri* extract on diabetic wound healing in rats. Specifically, we investigated the efficacy of combining *D. aucheri* extract with CMC gel dressing in enhancing wound closure, assessing both macroscopic and microscopic parameters of skin wound repair in a diabetic model.

## 2. Materials and Methods

### 2.1. Materials

Leaves, flowers, and stems of *D. aucheri* were collected from around Yasuj at flowering (March 2022). The plant was identified at the Herbarium of the Biosciences Department of Shahid Beheshti University (Tehran). The streptozotocin (STZ) and CMC were purchased from Sigma-Aldrich Co. The anesthetic agents (ketamine–xylazine) were purchased from Alfasan Co. (Woerden, Netherlands).

### 2.2. Animals Used in Experiments

This is an experimental study using interventional procedures on animals. This investigation involves 48 randomly selected Sprague-Dawley rats (2.5–3 months, 250 ± 10 g) that were prepared from Shiraz University of Medical Sciences. The animal chamber was maintained at a temperature of 20°C–22°C, with a photoperiod of 12 h of light and 12 h of darkness, and relative humidity ranging from 40% to 60%. The rats were housed in polycarbonate enclosures featuring a steel mesh top, with their bedding consisting of sawdust and wood chips. The cage flooring was replaced daily, and the cages were cleaned weekly.

### 2.3. Diabetes Induction

In the present study, diabetes was induced intraperitoneally in overnight-fasted male Sprague-Dawley rats through injection of 60 mg/kg body weight of freshly prepared STZ (Sigma-Aldrich, USA) dissolved in a 0.1 mol/L citrate buffer (pH 4.5) [36–39]. To confirm the induction of diabetes 7 days after STZ injection, the blood sugar of the animals was measured in mg/dL using a glucometer. If the blood sugar was higher than 300 mg/dL, the animals were considered diabetic and included in the study [38, 40]. The study's procedures were authorized by Shiraz University of Medical Sciences' Institutional Animal Ethics Committee (IR.SUMS.AEC.1401.102), in accordance with NIH publication no. 85-23, which was amended in 1996 and contains standards for the care and use of animals.

### 2.4. Determination of Total Phenols and Flavonoids of Hydroalcoholic Extract of *D. aucheri*

The total phenolic content of the extract was measured using the Folin–Ciocalteu method [19]. In this assay, phenolic compounds are oxidized in an alkaline medium containing the molybdotungstophosphoric heteropolyanion reagent, which initially has a yellow color. The reaction produces a blue-colored complex, the intensity of which depends on the phenolic composition and the pH of the solution, adjusted with sodium bicarbonate or sodium carbonate. For the assay, 0.2 mL of methanol solutions of different concentrations of each sample and gallic acid standard were mixed with 2 mL of 1:10 diluted Folin–Ciocalteu reagent. After 5 min, 1.5 mL of saturated sodium bicarbonate solution (60 g/L) was added. Following a 90-min incubation, the absorbance was measured at 725 nm using a spectrophotometer [41]. A standard curve was constructed using gallic acid at concentrations of 0, 25, 50, and 100 mg/mL, and the total phenolic content was expressed as gallic acid equivalents (GAE, mg gallic acid/g sample).

Using the calibration curve, the total phenolic content of the 80% methanol extract was determined to be 13.2 mg GAE/100 g of sample.

The total flavonoid content of the extract was determined using the aluminum chloride colorimetric method. In this assay, 500 μL of each extract was mixed with 1.5 mL of 80% methanol, 100 μL of 10% aluminum chloride solution, 100 μL of 1 M potassium acetate, and 2.8 mL of distilled water. After incubation for 40 min, the absorbance was measured at 415 nm against a control [33]. A standard curve was prepared using quercetin, and the flavonoid content of the extract was expressed as milligrams of quercetin equivalents per gram of dry weight (mg QE/g DW).

Based on this assay, the total flavonoid content of the extract was found to be 1.53 mg QE/g DW.

### 2.5. Preparation of Gel From Hydroalcoholic Extract of *D. aucheri* and 2% CMC Gel

In March 2022, we obtained specimens of *D. aucheri* with specimen code AR337E in the herbarium of the Biosciences Department of Shahid Beheshti University (Tehran) from Yasuj Province, Iran [34]. Aerial parts were cleaned and air-dried. Then 100 g of its powdered form was placed in a percolator and soaked in 500 cc of 70% ethanol for 72 h. The resulting solution, containing the hydroalcoholic solvent and extract, was then collected from the percolator. Using a rotary evaporator, the excess ethanol was completely removed, leaving a concentrated extract. The extract was then dried into a brown powder using a desiccator and a vacuum pump. Finally, based on the required dosages, the necessary amounts were measured. In order to facilitate the application of the agent, we provided 2%, 5%, and 10% *Dorema* (2, 5, and 10 g *Dorema* in 100 cc 2% CMC, respectively) [42–44]. The gel base (vehicle) was also supplied by creating 2% CMC (2 g CMC dissolved in 100 cc distilled water [45, 46]) gel without the *Dorema* component.

### 2.6. Animals and Excision of the Diabetic Wound Model

Diabetic rats were first subjected to general anesthesia by intraperitoneal injection of ketamine 100 mg/kg and xylazine hydrochloride 10 mg/kg. The hair on the back of their necks was shaved, and the area was disinfected with 70% ethanol. After anesthetizing the rats, we shaved the hair of the rats' backs, and a full-thickness circular wound with a diameter of 1.5 cm was created on the back of each animal using a sterile biopsy punch. On Days 0, 3, 7, and 14, photographs were taken of the wound healing process. Based on the following equation, the percentage of wound closure was calculated for each wound [39, 47, 48].

The wound closure rate was calculated as

(1) $$\begin{array}{c} \text{Percentage of wound healing}=\left(\frac{\text{Wound size in }a\text{ specific day}}{\text{wound size in Day }0}\right)\times 100. \end{array}$$

### 2.7. Experimental Design

The animals were divided into the following groups randomly:

  Healthy control group: In this group, only one wound with a diameter of 1.5 cm was created on the back of the animals (without any treatment).
  Diabetic control group: In this group, the animals became diabetic with a single intraperitoneal injection of STZ (60 mg/kg); then, a wound with a diameter of 1.5 cm was created on the back of the animals (without any treatment).
  Experimental groups 1 to 4: In this group, a wound with a diameter of 1.5 cm was created on the back of the animals, and CMC base gel (DM + B.g group), CMC + 2% hydroalcoholic extract (DM + *Dorema* 2% group), CMC + 5% hydroalcoholic extract (DM + *Dorema* 5% group), and CMC + 10% hydroalcoholic extract (DM + *Dorema* 10% group) were placed on the wound area, respectively, for 14 days [42–44].

After the end of the treatment period, the animals were anesthetized with a mixture of 100 mg/kg ketamine and 10 mg/kg xylazine (Alfasan Company, Netherlands); after Day 14, full-thickness skin biopsies (diameter 1.5 cm) were taken from the wound sites and fixed in buffered formaldehyde 10% (pH = 7.2) for stereological analysis.

### 2.8. Stereological Studies

The repaired area (equal to the original diameter) was excised using a biopsy punch. Then, the separated skin was placed in containers containing 10% formalin buffer solution (fixative) to conduct stereological studies. To create systematic, random, and uniform cuts (so that each part of the tissue has a chance to be selected once), we used the vertical uniform random (VUR) method. In this method, the piece obtained from the skin punch biopsy was cut vertically into small pieces; then, the pieces were removed one by one so that, finally, between 8 and 12 small pieces were selected. For conducting stereological studies, slices with a thickness of 5 and 20 μm were prepared from them, and the corresponding slides were prepared using hematoxylin–eosin and Masson's trichrome staining.

#### 2.8.1. Calculation of Volume Density

**Figure 1:**
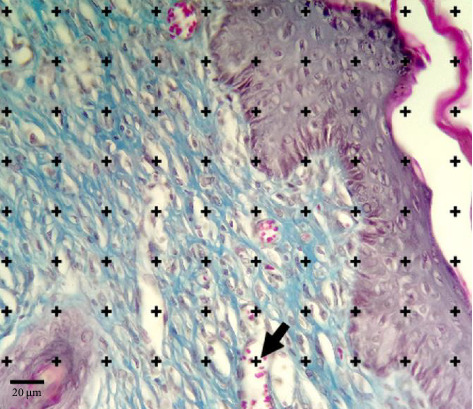
Grid point to calculate the volume density with the cross-point counting method, Masson's trichrome staining, magnification × 400. Conventionally, the areas that collide with the upper and right + parts are counted (arrow).

Using stereological methods, we measured the volume density of re-epithelization, collagen deposition, neovascularization, granulation, hair follicle and inflammation through the method of counting cross-points according to the Delesse formula (Figure 1). In this method, 5-µm sections were used to measure the volume density of a structure in the skin. A grid of intersecting points was placed over the microscopic image of the tissue, and the volumetric density of each parameter was determined using the following formula by counting the points that intersected with the structures of interest. For examining the average inflammatory cells and the volume density of the inflamed area, the number of points intersecting with inflammatory regions or newly formed blood vessels was counted and then divided by the total number of points intersecting with the tissue. This ratio was used to calculate the volumetric density of the parameter.

Then, the volume density of each part was calculated based on the following formula:

(2) $$\begin{array}{c} Vv\left(\text{structure}\right)=\frac{\sum_{i=1}^{n}p\;\left(\text{structure}\right)}{\sum_{i=1}^{n}p\;\left(\text{reference}\right)}. \end{array}$$

*p* (structure) is the number of points that collided with different parts of the skin tissue, and *p* (reference) is the total number of points that collided with the entire skin tissue.

#### 2.8.2. Numerical Density

**Figure 2:**
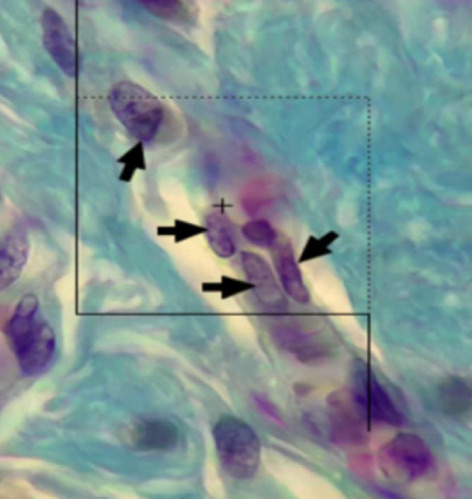
Counting the number of fibroblast cells using the optical dissector method, Masson trichrome staining, and magnification × 1000. Arrows indicate fibroblast cells.

Twenty-micron sections, stereology software, a Nikon microscope (e200 model), and a microcator (Heidenhain Mt12, made in Germany) were used to count the number of fibroblast cells. In this way, the unbiased counting frame in the stereology software was randomly placed on the images of the skin on the monitor; then, the average level of 10–80 microscopic fields was selected for each sample, and the number of fibroblast cells was counted using the optical dissector method. The micrometer was calculated at the height of the dissector (at a depth of 5–15 microns). To avoid strain, we counted all cells by shape and size. To measure the number of fibroblasts, we moved the optical section down in the *Z* axis; for air, a guard zone of 5 microns in the upper part and a guard zone of 5 microns in the lower part of the section were considered. Any cell that could be seen at the height of the dissector and was in the counting frame or in contact with the accept line was selected (Figure 2); then, the numerical density per volume unit was calculated using the following formula:

(3) $$\begin{array}{c} Nv=\frac{\sum_{i=1}^{n}Q}{\sum_{i=1}^{n}P\times h\times \left(a/f\right)}\times \frac{t}{BA}. \end{array}$$

In this formula, ∑*Q* is the number of fibroblast cells counted at the height of the dissector, ∑*p* is the total number of fields in which the counting was done, *a*/*f* is the area of the counting frame in all microscopic fields, *h* is the height of the dissector, *t* is the average slice thickness in different sections, and *BA* is the total thickness of the cut.

Any cell that could be seen at the height of the dissector and was in the counting frame or in contact with the accept line (dotted lines) was selected. Also, any cell outside the counting frame or in contact with the reject line (continuous lines) was not counted (arrow marks indicate the fibroblast cells).

### 2.9. Statistical Analysis

SPSS Inc., United States, Version 22 software, was used to analyze the data. The one-sample Kolmogorov–Smirnov test was used to evaluate the normality of data distribution. If the data had a normal distribution, one-way analysis of variance (ANOVA) and the LSD post hoc test were used for analysis. The Kruskal–Wallis test was used to analyze abnormally distributed data. Finally, the values were presented as mean ± SD, and a *p* value less than 0.05 was considered significant. The relevant histograms were drawn using GraphPad software Version 7.8.

## 3. Result

### 3.1. Comparison of Body Weight and Fasting Blood Glucose

The weight of the animals in the study groups and their fasting blood sugar (FBS) levels after 7 days of intraperitoneal injection of streptozotocin are summarized in Table 1. There were no statistically significant differences between the groups in body weight. The result showed a significant increase in the weight of all diabetic groups compared to the control group.

### 3.2. The Macroscopic Area of the Wound Results

**Figure 3:**
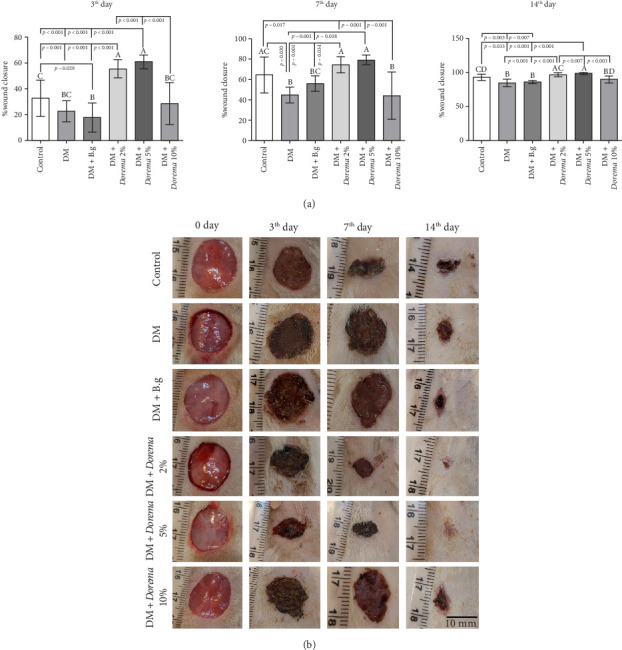
Photograph of macroscopic images of the wound healing process on Days 0, 3, 7, and 14 in different groups.

Macroscopic observation of wounds and their imaging was done on Days 0, 3, 7, and 14 in the study groups. The results showed that on the 3rd day, in the groups that received 2% and 5% *D. aucheri* extract hydrogel, the highest percentage of wound healing was observed, while in the DM and diabetic + base gel groups, the lowest healing rate was observed (*p* ≤ 0.05). The highest percentage of wound healing was observed on the seventh day in the groups that received 2% and 5% *D. aucheri* plant hydrogel, compared to the control, diabetic, and diabetic + base gel groups. Similarly, the highest percentage of wound healing on the 14^th^ day was also related to the group that received *D. aucheri* plant extract 2% and 5% compared to the diabetic and diabetic + base gel groups (*p* ≤ 0.001) (Figure 3).

### 3.3. Stereological Evaluation

**Figure 4:**
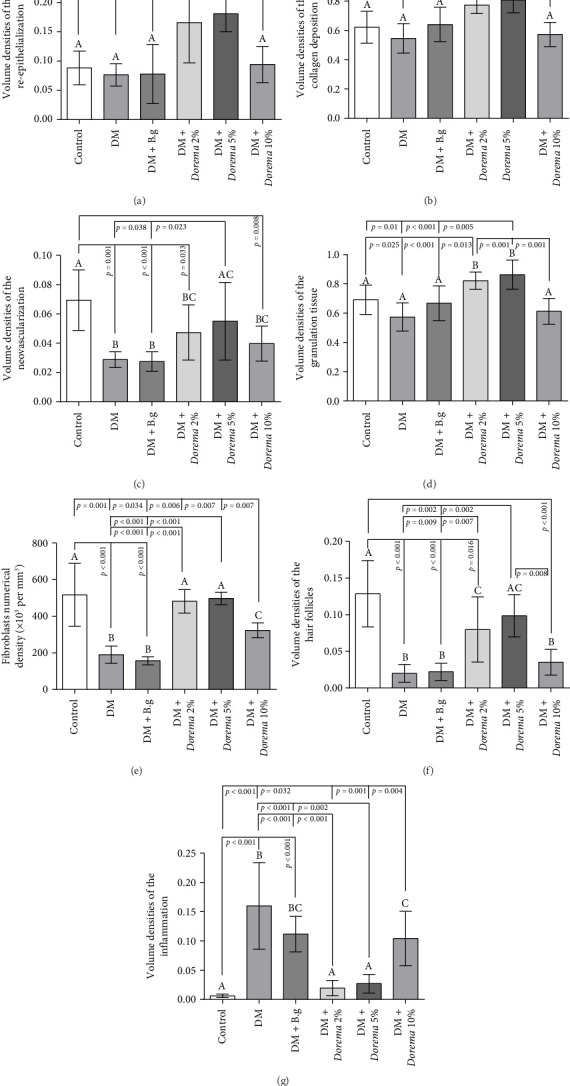
Comparison of the mean stereological results, assessing the volumetric density of (a) re-epithelialization, (b) collagen deposition, (c) neovascularization, (d) granulation tissue formation, (e) fibroblast numerical density per unit volume, (f) hair follicle density, and (g) inflammation levels on Day 14 across the different study groups. Values are reported as mean ± SD. (a, b, c) There was no significant difference between the columns with at least one similar letter. However, dissimilar letters indicate a significant difference (*p* ≤ 0.05). Control (*n* = 6), DM (*n* = 4), DM + B.g (*n* = 5), DM + *Dorema* 2% (*n* = 6), DM + *Dorema* 5% (*n* = 4), DM + *Dorema* 10% (*n* = 5).

The volume density of the re-epithelialization, collagen deposition, and the volume of the granulation tissue were higher in the DM + *Dorema* 5% and DM + *Dorema* 2% groups, which were statistically significant compared to other study groups (*p* ≤ 0.05 and *p* ≤ 0.001), respectively (Figures 4(a), 4(b), and 4(d)). Some collagen depositions after 14 days were observed in the CMC base gel group; however, the changes were not significant compared to the control and diabetic control groups (Figure 4(b)).

The average volume density of new blood vessel formation (neovascularization) and hair follicles in the control group was higher than the other groups (except DM + *Dorema* 5%), (*p* < 0.05) (Figures 4(c) and 4(f)). The results showed a significant increase in the volume density of the hair follicles in DM + *Dorema* 5% and the DM + *Dorema* 2% groups compared to the DM and DM + B.g groups (*p* < 0.05) (Figure 4(f)).

The results of the optical dissector and numerical calculation of the fibroblast cells in different groups showed that the lowest number of cells in volume density was observed in the DM and DM + B.g groups compared to the control group (*p* < 0.001). In the DM + *Dorema* 5% and DM + *Dorema* 2% groups, the density of the fibroblast cells indicated a significant increase compared to the DM and DM + B.g groups (*p* < 0.001). The number of fibroblast cells in the DM + *Dorema* 10% group showed a significant increase compared to the DM and DM + B.g groups; however, it was not the same as the DM + *Dorema* 5% and DM + *Dorema* 2% groups, so it also showed a significant difference compared to them (*p* < 0.05). These results are shown graphically in Figure 4(e).

Examining the average inflammatory cells and the volume density of the inflamed area showed that the highest amount of inflammation was observed in the diabetic control group, the DM + B.g group, and the DM + *Dorema* 10% group, respectively. These changes were significant compared to the other groups. However, in the DM + *Dorema* 2% and DM + *Dorema* 5% groups, the changes were not significant compared to the control group despite the presence of a little inflammation (Figure 4(g)).

### 3.4. Histopathological Results

**Figure 5:**
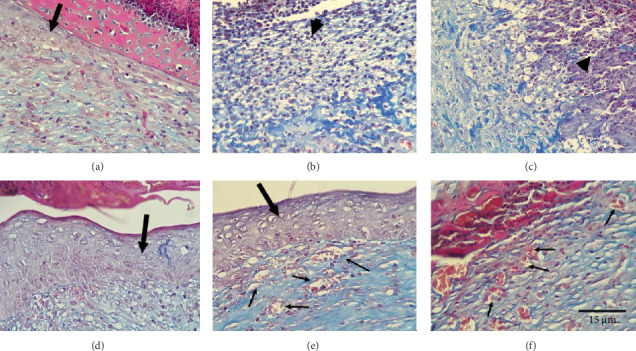
Light photomicrograph of the wound site in the tested groups: (a) healthy control group; (b) diabetic control group (DM); (c) diabetic group + base gel (DM + B.g); (d) DM + *Dorema* 2%; and (e) DM + *Dorema* 5%. Neovascularization was observed in different groups (small arrows). On the other hand, relatively re-epithelialized tissue can be seen on the surface of the wound (big arrow). Arrowhead indicates inflammation. In the group that received the 5% dose, a relatively more effective function was observed (f): DM + *Dorema* 10% (Mason trichrome staining, × 400 magnification).

The results of Masson's trichrome staining of the wound site in different groups studied on the 14th day showed that in the control group, the diabetic control group, and the CMC group alone, there was a low density of collagen fibers and edema. It was observed under the clot, and the covering tissue was incomplete. However, in other groups, collagen fibers and pericell granulation tissue indicated proper healing of the wound area. In the control group, inflammatory exudate and non-migration of the spiny cells and pericellular granular tissue were observed. In this group, the volume ratio of hair follicles showed a significant increase compared to other groups. Collagen fibers and angiogenesis in the treatment groups were more regular and wider than in the control group. In comparison with the control group, the restoration of the epidermal tissue was very clear and prominent, and the sebaceous glands and hair follicles were forming. As shown in the diabetic control group, inflammatory exudate and lack of migration of spiny cells, a significant reduction of angiogenesis, and formation of bud tissue were observed. It can be seen under the scab. In other words, the granule tissue was less stringy. A significant decrease in angiogenesis and the formation of budding tissue was observed under the low stringy scab, as shown in group C. In the DM + *Dorema* 5% group, the wound space was filled by granulation tissue that had reached maturity, contained relatively dense collagen fibers, and was mature. Also, a significant increase in the number of fibroblasts and angiogenesis was shown. As shown in the DM + *Dorema* 10% group, the granular tissue, lower filaments, and the rate of re-epithelialization did not show any significance (Figure 5).

## 4. Discussion

In the present study, the healing effects of the alcoholic extract of the *D. aucheri* plant in the form of 2%, 5%, and 10% gels with CMC based on full-thickness wounds in the diabetic animal model of rats were investigated. Plants are traditionally used in the treatment and prevention of various disorders [49, 50]. The results of the macroscopic and microscopic evaluations showed that the *D. aucheri* plant extract effectively increased the contraction on the wound surface, decreased the presence of inflammatory cells, strengthened the process of cell repair, and increased the density of collagen and healing rate on all days of the study. Based on the macroscopic examination, the use of 2% and 5% *D. aucheri* plant extract improved diabetic wound healing better than 10% extract. This could be because higher percentages of porosity prevent the formation of blood vessels by reducing the amount of porosity in the base gel; also, when forming and sprouting new vessels are reduced, the fibroblast cells are unable to enter the new tissue and begin to form collagen. The stereological results of the number of fibroblasts and the density of blood vessels and collagen fibers confirmed this issue.

Bilhar leaf hydroalcoholic extract protects liver function in diabetic rats. These effects are also due to the presence of flavonoids in the extract of Bilhar leaves. Previous studies have shown that in wounds, the 7th day, due to the overlap between the inflammatory and proliferative phases, and the 14th day, due to the overlap between the proliferative and regeneration phases, are the most appropriate times to investigate the reparative effects of the compounds used in wound healing. It has been described in various articles that *Dorema* has the property of healing wounds and lesion areas. Therefore, it was especially used for vitiligo, melasma, and freckles [51, 52]. In our study, the use of this alcoholic extract improved the lesion area.

In the present study, angiogenesis was evaluated using the stereological method. Stereological studies showed a significant number of fibroblast cells and an increase in the volume of the epidermis and dermis in the groups treated with the extract compared to the diabetic control groups. On the other hand, angiogenesis is one of the most important factors in the process of optimal wound healing. The process of angiogenesis can be accelerated due to the presence of growth factors such as TGF-β and the reduction of inflammation [53]. Studies by other researchers also show that *D. aucheri* plant extract contains flavonoids, so it has anti-inflammatory and pain-relieving effects [54]. Also, coumarins and terpenes can be mentioned among other compounds of this plant, which inhibit the release of neurotransmitters from the terminals of pain fibers in the posterior horn of the spinal cord and ultimately reduce pain [55].

Also, in this study, the collagen density of the dermis was evaluated as one of the most important indicators of tissue maturity, along with other stereological characteristics. Also, Masson's trichrome staining was used to evaluate the collagen density in the restored dermis. Collagen, as the most abundant protein in the body, is one of the most important secretions of the fibroblasts, whose production and regeneration play an essential role in the wound-healing process. The results of the present study showed that the highest number of fibroblast cells and the highest amount of collagen were observed in the control and 5% and 2% gel groups. In some studies, high concentrations of this plant have had toxic effects on the cells; perhaps, for this reason, the wound-healing process has been slower [56, 57].

The extract of the *D. aucheri* targets several pathways involved in the healing process of diabetic wounds through its bioactive compounds. This plant contains bioactive constituents such as various phenolic acids, flavonoids, coumarins, sesquiterpenes, and terpenoids [49]. Among other compounds present in this plant are carvacrol and thymol, which exhibit antioxidant [30, 58], anti-inflammatory, antimicrobial [59], and tissue-repairing effects that are vital for the improvement of diabetic wounds [60]. It has been demonstrated that carvacrol reduces the concentration of TNF-α [61, 62]. The activation and stimulation of macrophages and endothelial cells following oxidative stress induced by hyperglycemia lead to the activation of IκB kinase (IKKβ) and the transcription factor NF-κB, resulting in an increased secretion of TNF-α [4]. Consequently, these factors contribute to an increased volumetric density of inflammatory regions, prolonged inflammatory responses, and delayed wound healing. Furthermore, TNF-α disrupts the tissue remodeling process in diabetic individuals by inducing apoptosis in fibroblasts and inhibiting collagen synthesis through reduced hydroxyproline production. This inhibition of keratinocyte migration delays epithelialization and the wound healing process [61–63]. Therefore, reducing TNF-α enhances the wound healing process. Recent studies have also shown that certain phytochemical compounds, due to their antioxidant properties, can inhibit the secretion of IL-6 and TNF-α in diabetic wounds by reducing reactive oxygen species (ROS) [64–66]. The flavonoid and alkaloid compounds in the extract of *D. aucheri* are recognized as anti-inflammatory and antioxidant agents that can inhibit ECM degradation by matrix metalloproteinases (MMPs) and enhance the expression of collagen synthesis-related genes such as COL1A1 and COL3A1. Additionally, the activation of fibroblast growth factor (FGF) and increased production of fibronectin and laminin may improve ECM structure and expedite epithelial and vascular tissue reconstruction. Moreover, it has been shown that carvacrol activates vascular endothelial growth factor (VEGF), leading to increased angiogenesis for improved blood flow at wound sites [62]. This provides evidence for the increased volumetric density of neovascularization, granulation, and re-epithelialization observed in groups treated with 2% and 5% doses of *D. aucheri* extract.

In a study by Eftekhari et al. on the toxic effects of *D. aucheri* on fibroblast cells, it was concluded that the compounds of *D. aucheri* had the potential to exert toxic effects on healthy cells. However, a negative correlation was observed between cytotoxicity and the phenol content of *D. aucheri* fractions [31]. In another study, twenty-five compounds were identified in the essential oil of *D. aucheri*, with the major constituents being caryophyllene (E) (31.29%), phytol (14.92%), β-gurjunene (9.84%), 3,7,11,15-tetramethyl-2-hexadecen-1-ol (8.7%), and *n*-hexadecanoic acid (8.09%). The MTT assay determined the IC50 values of the essential oil to be 1.4 mg/mL for the SW48 cell line and 1.2 mg/mL for the SW1116 cell line. Flow cytometry analysis revealed that the essential oil significantly increased apoptosis in the SW48 cell line compared to vincristine (*p* < 0.05). Although apoptosis also increased in SW1116 cells compared to vincristine, the difference was not statistically significant. These findings indicate that the essential oil of *D. aucheri*, which contains high levels of caryophyllene, exhibits significant cytotoxic effects against SW48 and SW1116 cancer cell lines [67].

Mostafavi et al. demonstrated in their research that the injection of *D. aucheri* extract leads to necrosis, inflammation, and elevated liver injury markers (ALT and AST) at high doses. This toxicity is likely attributable to the plant's bioactive compounds, which disrupt metabolic liver function [68]. It is possible that the sesquiterpenes present in this plant, at elevated concentrations, cause the leakage of cytochrome c from mitochondria and activate apoptosis [69]. Additionally, the flavonoids in *D. aucheri* extract may induce apoptosis in fibroblasts in diabetic mice by activating the caspase-3 pathway at high doses, as it has been established that flavonoids exhibit cytotoxic effects at elevated concentrations [70]. This highlights the necessity for further studies in this area. Plants contain compounds such as alkaloids, mycotoxins, genotoxins, or heterocyclic amines (HCAs) that can damage DNA or induce oxidative stress. These compounds may be present in the *D. aucheri* extract [68]. Therefore, there is a need for more extensive research and phytochemical analysis to investigate the effects of these compounds at varying doses. In fact, certain phytochemicals may disrupt the oxidative-antioxidative balance of cells at high doses by generating free radicals, leading to damage to cellular or mitochondrial membranes. They may also activate signaling pathways associated with apoptosis or necrosis.

To the best of our knowledge, this study was the first one evaluating the effect of different concentrations of *D. aucheri* plant extract on diabetic wound healing, using stereological studies. However, there are some limitations. Cytotoxicity studies and biochemical mechanisms of wound healing were not evaluated in this study. Further research is recommended to include evaluations of the mechanisms of action, cytotoxicity studies, and mechanical assessments such as the tensile strength test, adhesion strength test, viscoelastic properties, punch test, torsional test, and non-invasive techniques (cutometer, durometer, and ballistometer), as well as the peel test and compression test. The research was conducted on a rat model, and the extrapolation of these results to human diabetic wounds may not be straightforward due to interspecies variations in wound-healing processes. Future studies on diabetic wound healing of *D. aucheri* plant extract are proposed on humans. Given the widespread use of *D. aucheri* as a traditional herbal remedy in certain regions of Iran, further studies are essential to identify its potentially toxic constituents and clarify its molecular mechanisms of action. This includes phytochemical analysis and evaluation of long-term effects. The toxicity of *D. aucheri* extract at high doses is likely due to bioactive compounds that may be beneficial at lower concentrations. Therefore, determining a safe dosage and achieving a balance between therapeutic efficacy and toxicity is critical for clinical use. Additional pharmacokinetic and clinical studies are needed to define a safe dosing range.

## 5. Conclusion

This experimental study demonstrated that the 2% and 5% concentrations of *D. aucheri* (Bilhar) extract significantly enhanced the wound healing process. The extract promoted wound contraction, reduced inflammation, and improved collagen density and fibroblast proliferation, suggesting its potential therapeutic application in diabetic wound healing. However, further studies, particularly clinical trials, are suggested to be conducted to confirm its efficacy and safety in human subjects.

## Figures and Tables

**Table 1:** Body weight and fasting blood sugar 7 days after streptozotocin injection in the studied groups.

Groups	Body weight (g)	FBS (mg/dL)
Control	294.28 ± 22.43^a^	87.71 ± 6.26^a^
DM	294.20 ± 16.44^a^	472.00 ± 88.60^b^
DM + B.g	296.80 ± 14.49^a^	463.40 ± 50.81^b^
DM + *Dorema* 2%	288.40 ± 27.80^a^	483.12 ± 65.51^b^
DM + *Dorema* 5%	292.60 ± 19.74^a^	470.60 ± 79.48^b^
DM + *Dorema* 10%	295.90 ± 17.37^a^	467.70 ± 71.59^b^

*Note:* (a, b) There was no significant difference between the columns with at least one similar letter. However, dissimilar letters indicate a significant difference (*p* ≤ 0.05).

## Data Availability

This article contains all the data created and examined throughout this investigation. The corresponding author will provide the datasets used or analyzed during the current work upon reasonable request.

## Nomenclature

CMC: Carboxymethyl cellulose
STZ: Streptozotocin
B.g: Base gel
DM: Diabetes
VUR: Vertical uniform random
SPSS: Statistical Package for the Social Sciences
ANOVA: One-way analysis of variance
FBS: Fasting blood sugar
SD: Standard deviation
